# Editorial: Exploring GWAS data by biomolecular network analysis in revealing genetic disease mechanisms

**DOI:** 10.3389/fgene.2023.1223913

**Published:** 2023-05-30

**Authors:** Nicholas K. Moschonas, Maria I. Klapa

**Affiliations:** ^1^Laboratory of General Biology, School of Medicine, University of Patras, Patras, Greece; ^2^Metabolic Engineering and Systems Biology Laboratory, Institute of Chemical Engineering Sciences, Foundation for Research and Technology-Hellas (FORTH/ICE-HT), Patras, Greece

**Keywords:** genome-wide association studies, biomolecular interaction networks, regulatory networks, pathway analysis, disease genetic architecture, network biology

## 1 Introduction

Since the publication of the first draft of the human genome sequence in 2001 and especially within the past decade, genome-wide association studies (GWAS) have largely contributed in accelerating the identification of genetic variants across the genomes of millions of individuals, statistically associated with various specific traits and disease phenotypes (e.g.,: Uffelmann et al., 2021). For common diseases, e.g., type 2 diabetes mellitus (e.g., Mahajan et al., 2022), coronary artery disease (e.g., Musunuru and Kathiresan, 2019), schizophrenia (e.g., Kato et al., 2023), lung cancer (e.g., Long et al., 2022), thousands of susceptibility genetic loci have been determined, the analysis of which may reveal valid functional information about the disorders, accelerate drug development, and contribute to the design of predictive health diagnostics. However, the effect size of each individual variant associated with a multifactorial disease is relatively small, the overall disease risk corresponds to the cumulative effect size resulting from multiple genes and their interactions. The information content of GWAS data can be upgraded in the context of biomolecular interaction networks, selectively or in combination, e.g., protein interaction, metabolic, RNA and/or gene regulatory networks (e.g., Tomkins and Manzoni, 2021; Farrow et al., 2022). Such strategies have successfully been used for the evaluation of GWAS combined with relevant gene expression data (e.g.,: Wainberg et al., 2019) to a) prioritize groups of core susceptibility genes with respect to their position in the biomolecular network (Zhang et al., 2021), b) reveal disease modules, pathways, and disease subgroups (e.g., Wu et al., 2021), c) discover novel, and extent currently known disease-associated biological mechanisms and pathways (e.g., Wang et al., 2020), d) identify additional risk genes based on the network architecture of the disease (e.g., Meng et al., 2020), and e) suggest repurposing or development of new drugs to plausible targets through disease comorbidity assessment (e.g., Reay and Cairns, 2021; Barrio-Hernandez and Beltrao, 2022). Integration of GWAS data from various studies and experimental platforms including SNP arrays, exome or whole genome sequencing or single-cell omics, increases the number of identified genetic risk variants, enhancing thus our knowledge about disease/trait heritability (e.g., Wang et al., 2022). Integrated datasets widen the risk allele frequency spectrum, and may contribute to the identification of causal gene mutants for rare diseases. GWAS integrated with genome-wide transcriptomics data subjected to biomolecular network and pathway analysis would permit the quantitative functional profiling of genetic variants and may suggest novel disease-associated biological mechanisms and regulatory structures.

Six original research articles covering various of the aforementioned areas have been included in this Research Topic.

Rahmouni et al. identified novel biological pathways and genes associated with four main characteristics of skin aging by analyzing relevant GWAS genotyping data of 502 Caucasian women. Data integration into the reference KEGG pathways and search for associations with significant pathways by gene set enrichment analysis, revealed the “nucleotide excision repair”, the “mTOR” and the “proteasome” pathways involved in DNA maintenance, protein integrity, and cell survival. *XPC*, *AD23B*, and *DDB1* are among top-ranked genes with the latter possibly involved in photoaging, sagging, and wrinkling in response to UV exposure. In addition, the “melanogenesis” pathway was revealed, a skin aging-specific pathway, with *WNT7B* involved in Wnt/β-catenin pathway playing critical roles in embryogenesis and adult tissue homeostasis, and *PRKCA* involved in regulation of cell proliferation, apoptosis, differentiation, adhesion, and tumorigenesis. According to the authors some of the indicated pathways may participate in aging characteristics of other organs, as well.

Azumah et al. associated the expression pattern of polycystic ovary syndrome (PCOS) candidate genes during fetal development with potential upstream regulators and related pathways. A supervised heat map of RNA sequencing data correlated the expression pattern of PCOS genes with the gestational age. Ingenuity and conventional KEGG pathway analysis revealed several key pathways associated with PCOS and co-expressed genes. The early-expressed gene cluster was found involved in mitochondrial function and oxidative phosphorylation with upstream regulators including *PTEN*, *ESRRG*, *ESRRA*, and *MYC*. The late-expressed gene cluster was found associated with stromal expansion, cholesterol biosynthesis and steroidogenesis with upstream regulators including *TGFB1*, *TNF*, *VEGF*, *ERBB2*, and *ERBB3*. Noteworthy, many of the suggested upstream regulators like *MYC*, *ERBB2*, *TNF*, and *TGFB1* are genes with high network connectivity, involved in several interconnected essential pathways.

Chen et al. have proposed an integrated procedure for the identification the genes that participate in tumor initiation (“leader genes”) in stomach, liver and colon cancer, contributing to the elucidation of the mechanisms and early diagnosis of cancer. Their approach included information derived from node interconnectivity based on validated protein-protein interaction networks, literature data on cancer-related characteristics and experimental proteomic data including subcellular localization information. A graph theory-based algorithm was proposed to reveal and validate candidate leader genes. *TRIP13*, a gene playing a key role in chromosome recombination and chromosome structure development during meiosis, was identified as a common leader gene. Overall, the authors’ lists of leader genes for stomach, liver and colon cancer included 69, 43, and 64 genes, respectively.

Meng et al. integrated transcriptomic with GWAS data in order to determine new biomarkers associated with atrial fibrillation (AF). More specifically, they employed weighted gene co-expression network analysis (WGCNA) to identify significant network modules related to AF and fuse this information with the GWAS discovery set produced from the GWAS data from ProxyGeneLD. Two genes, *ERBB2* and *MYPN*, emerged as most associated with AF occurrence and form the basis for further investigation.

Chimusa and Defo introduced the ancMETA tool, which introduces a Bayesian graph-based framework connecting SNPs to genes and genes to PPI networks, identifying the interaction pathways underlying a pathophysiology across multiple ancestries. The framework was evaluated based on simulated datasets and demonstrated in the context of seven European bipolar disorder cohorts.

In Dong et al. a weighted correlation network analysis was performed on the integrated mRNA, miRNA and lncRNA datasets of ulcerative colitis to reconstruct the competitive endogenous RNA (ceRNA) network in the disease. The ceRNA network study through Gene Ontology (GO), pathway enrichment and PPI network analysis lead to the identification of two mRNAs as high diagnostic accuracy biomarkers, providing also insight into the pathogenesis of the disease.
